# Factors associated with morbidity of a totally implantable venous access device in patients with breast cancer

**DOI:** 10.55730/1300-0144.5904

**Published:** 2024-09-20

**Authors:** Burak İLHAN, Berkay KILIÇ

**Affiliations:** 1 Division of General Surgery, Department of Surgery, Faculty of Medicine, İstanbul University, İstanbul, Turkiye; 2 Division of General Surgery, Department of Surgery, Institute of Oncology, İstanbul University, İstanbul, Turkiye

**Keywords:** Totally implantable venous access device, port catheter, breast cancer, morbidity

## Abstract

**Background/aim:**

To investigate the possible morbidities associated with the implantation of a totally implantable venous access device (TIVAD) in breast cancer (BC) patients.

**Materials and methods:**

Clinical data and developed complications in 546 BC patients with TIVAD between 2017 and 2021 were analyzed retrospectively. Among these, 524 (96%) patients who underwent TIVAD implantation via the right subclavian vein (SCV) route were examined separately.

**Results:**

The mean patient age was 56.1 ± 11.4 years, and the mean patient body mass index was 25.3 ± 6.5. The incidence of early complications was 2.3% (12 cases), and the late complication incident rate was 2.6% (14 cases). Among these complications, ten (1.9%) catheter-related infections developed more frequently than others. Early complications increased as the number of puncture attempts rose and decreased with ultrasound guidance in the implantation, but no other predictive factors increased. No correlation existed between the patients’ age, body mass index, BC side, puncture method, puncture number, surgical approach, radiotherapy, and late complications. There was no evidence that postoperative effects specific to BC surgery, such as increased pain, decreased functional capacity, and lymphedema, were altered with TIVAD implantation. For catheter-related infections only, adjuvant radiotherapy was a risk factor for patients with various comorbidities.

**Conclusion:**

This study concludes that the right SCV approach remains a morbidity-safe route for TIVAD implantation in BC patients, as in other malignancies. The study suggests that clinicians can use other implantation routes to avoid catheter-related infection in patients scheduled for neoadjuvant treatment, who also have morbidity, and who are likely to receive postoperative radiotherapy.

## 1. Introduction

Breast cancer (BC) remains a significant public health problem. Despite current efforts to prevent the disease, its incidence is increasing in most countries. Chemotherapy is an essential part of treatment for the vast majority of BC patients. Totally implantable venous access device (TIVAD) systems allow permanent and safe central venous access to prevent recurrent thrombophlebitis or thrombotic events in the peripheral venous system that may restrict the infusion of various chemotherapy agents. They also enable the use of highly concentrated nutritional products in the presence of progressive disease [1].

Patients facing psychiatric problems potentially associated with having gynecologic cancer and those with cosmetic concerns may also experience issues such as pain, functional inadequacy, and lymphedema due to surgery; they may also develop additional complications related to port implantation. These complications can occur in the early period (pneumothorax, malposition hemothorax, accidental arterial puncture) or late period (catheter-related infection, thrombotic events, catheter fracture) following implantation. Many studies have investigated the complications related to TIVAD implantation, the potential risks of complications, and their prevention, but with conflicting results [2,3]. There are few studies on TIVAD implantation and complications specific to BC patients, and studies have limited sample sizes. Therefore, no reasonable guidelines or recommendations regarding complications related to TIVAD implantation in patients with BC have been established. Thus, this study aimed to investigate the possible relationships between morbidity and implanted TIVADs in patients with BC.

## 2. Materials and methods

### 2.1. General principles

This retrospective study analyzed the morbidity outcomes associated with TIVAD implantation of BC patients at the Department of Surgery at the İstanbul Faculty of Medicine and Oncology Institute from July 2017 to September 2021. Since the right subclavian vein (SCV) was the preferred insertion site in almost all patients in the study group, the outcomes of this implantation route were analyzed. The study was approved by the Ethical Committee (decision year/no: 28.09.2023/2137999) of İstanbul University’s Medical Faculty.

The inclusion criteria were: 1) BC patients undergoing breast surgery; 2) a need for adjuvant chemotherapy; and 3) patients sufficiently physically fit to tolerate the procedure. The exclusion criteria were: 1) vascular anatomic anomalies detected before the procedure; 2) signs of infection at the implantation site on physical examination; 3) anticoagulant therapy before the procedure or predisposition to thrombosis; and 4) various comorbidities that would prevent tolerance to the procedure.

Early complications were those observed intraoperatively or detected by control imaging immediately after the procedure. In contrast, late complications were those detected during or after ongoing chemotherapy regimens. Complications specific to BC surgery exist, such as arm and shoulder pain, functional disability, or lymphedema. To interpret the possible effects of axillary dissection on these types of complications, patients who underwent right-sided axillary dissection for right-sided TIVAD were compared with those who did not and patients who underwent left-sided breast surgery. To interpret the possible effects of adjuvant radiotherapy on these complications, patients who received right-sided lymphatic or breast radiotherapy were compared to those who did not.

### 2.2. Implantation procedures

After identifying the anatomical landmarks in the SCV localization in the implantation with conventional percutaneous or ultrasound (US)-guided techniques, the insertion site was sterilized with an alcoholic chlorhexidine solution. Local anesthesia was administered to the puncture and pocket sites of all patients. After a venous puncture, a guide wire was sent through the needle. An incision was made with the guide wire in the center, and the vascular dilator was advanced over the guide wire. After the guide wire was removed, a 7.5 F catheter was passed through the dilator into the subclavian vein and then into the cavoatrial junction, taking into account patient-specific characteristics. The most recently used technique is intracavitary electrocardiography for the consequent tip position near the cavoatrial junction. An incision of approximately 2–3 cm was made with the port pocket above the second costa, and a subcutaneous pocket suitable for the reservoir dimensions was created by blunt dissection caudal to the incision. The catheter was tunneled from the puncture site to the port pocket. The port chamber was placed in this pocket and fixed by holding sutures. After the procedure, an X-ray chest radiograph was taken to check port catheter placement and detect possible early complications. Patients were seen again for a follow-up 1 week after the procedure.

### 2.3. Statistical analysis

Data on the patients’ age, body mass index, placement side, insertion method, number of punctures, insertion method, surgical procedure, data of receiving adjuvant radiotherapy, and developed complications were collected. A Fisher’s exact test or the χ2 test in two-tailed univariate analyses was conducted. An independent t-test was used to determine associations between complications and the mean demographic data of patients. In addition, a stepwise backward logistic regression model was utilized to assess significant associations in the multivariate model, with developed complications set as the dependent variable. The results had a confidence interval of 95%, and p < 0.05 was considered statistically significant. SPSS, version 21.0 (IBM Corp., Armonk, NY, USA), was used in the statistical analyses.

## 3. Results

The demographic and clinical data of the patients with BC who underwent TIVAD implantation via the right-sided SCV route are presented in Table 1. The mean patient age of the group was 56.1 ± 11.4 years (range: 28 to 81 years). The mean patient body mass index was 25.3 ± 6.5 (range: 19 to 38). Five hundred and twenty-four (95.9%) TIVADs were placed right-sided and 22 (4.1%) TIVADs on the left side. Two hundred-eighty (53.4%) TIVADs were implanted ipsilaterally to the BC side and 244 (46.6%) to the contralateral side. In 96 (18.3%) implantations, puncture attempts were performed using classic anatomical landmarks, and in 428 (81.7%) implantations, US guidance was used. One hundred and eighty-two (34.7%) punctures were successful in the first attempt, 214 (40.8%) in the second, and 128 (24.5%) in the third or further attempts. In the group, 351 (66.9%) patients underwent breast-conserving surgery, and the remaining 173 (33.1%) patients had a mastectomy. While 106 (20.2%) TIVADs were implanted ipsilateral to the BC side in patients who underwent axillary dissection, 179 (34.2%) patients received right-sided adjuvant radiotherapy. In total, 26 (4.9%) patients developed complications. The incidence of early complications was 2.3% (12 cases), and for late complications, the rate was 2.6% (14 cases). Seven arterial punctures, three pneumothoraxes, and two malpositions were early complications. Ten catheter-related infections, three thrombotic events, and one catheter fracture (pinch-off syndrome) were late complications. Eight (1.5%) patients required port removal due to complications.

Although arterial puncture is a common complication that does not usually pose a significant problem with minimal compression, it has been known to impair coordination later in the procedure. Consistent with this, no arterial puncture in the study period required further intervention and disappeared with simple pressure application. One minimal pneumothorax resolved spontaneously. Two patients with pneumothoraxes required thoracic tube drainage with hospitalization, and these were resolved at the 5-day follow-up; subsequently, the drainage systems were removed. Two malpositioned catheters were rotated with the assistance of digital subtraction angiography and advanced into the proper location.

The most frequently observed complication was catheter-related infection. Six patients experienced pocket infections, and four suffered from port-related bacteremia. All these patients received antibiotics, and five (0.9%) needed catheter removal. The three patients with thrombotic events received low-molecular-weight heparin and oral anticoagulants for varying periods. Of these three patients, two needed catheter removal. One catheter fracture (pinch-off syndrome) was detected by control X-ray, and additional computed tomography after the infusion of the second chemotherapy regimen failed due to obstruction. The fractured part of the catheter was extracted from the right ventricle with the assistance of digital subtraction angiography, and the port was removed. A total of eight (1.5%) patients required TIVAD removal.

The patients’ age, body mass index, BC side, insertion method, breast surgery, axillary approach, and adjuvant radiotherapy did not increase the incidence of complications. The incidence of early and overall complications increased significantly with increasing punctures, whereas it decreased with US guidance. No factor influenced the incidence of late complications. Risk factor analysis for complications is presented in Table 2.

If catheter-related infections were evaluated in detail, the patients’ age, body mass index, TIVAD placement side, insertion method, number of puncture attempts, breast surgery, and axillary approach could not be associated with catheter-related infection risk. Two of the patients who developed catheter-related infections had diabetes mellitus, one had respiratory disease, and three had a history of smoking. In patients who received adjuvant radiotherapy, TIVAD implantation from the side ipsilateral to the BC side was associated with an increased incidence of catheter-related infections (p = 0.022). Multivariate analysis also showed that the risk of catheter-related infection increased in patients receiving adjuvant radiotherapy (p = 0.028) (Table 3).

The patient-reported rate of persistent breast, arm, and shoulder pain after BC surgery ± radiotherapy within one to three years was 33.7% in patients with TIVAD and 31.8% in patients without TIVAD. After one to three years following breast surgery, restriction of arm and shoulder movements occurred in 28.6% of the patients with TIVAD and 27.8% of those without TIVAD. The incidence of lymphedema was 15.7% in patients with TIVAD and 14.9% in patients without TIVAD. The incidence of breast surgery-specific complications was not affected by TIVAD implantation (p > 0.05).

## 4. Discussion

Patients with BC often require long-term chemotherapy and intermittent blood sampling, but the vast majority of chemotherapy agents have venous toxicity. Because of venous toxicity, thrombosis or thrombophlebitis in peripheral veins usually occurs, leading to an inability to maintain chemotherapy infusion and multiple venous punctures and adding another concern to the already anxious psychiatric state of some patients. In these circumstances, TIVAD stands out as a convenient and comfortable option that allows for easier chemotherapy cycles [4,5]. However, serious complications can still occur during TIVAD implantation and follow-up. Numerous studies have investigated TIVAD implantation procedures and possible complications, but conflicting results were obtained because the patient population may have included patients with varying demographics and malignancies. Some studies have associated the risk of infection with increasing age [6], while many others have revealed no correlation between patient age and infection rate [7]. In the present study, no relationship existed between the age of BC patients and complications. Several studies have reported that the risk of complications increases with a higher body mass index, while others have shown that patients with low body mass index are more prone to developing pneumothorax [8]. The current study found no such association between the body mass index of patients with BC and complications.

Evidence in the literature has shown that using US during insertion might lead to a decreased rate of complications [9]. We found a similar significant correlation in complication rates between US-guided punctures compared to punctures using anatomical landmarks. A lower early complication rate was achieved with US during the puncture, whereas late complications were not associated with the same procedure. Studies in the literature have also revealed that the complication rate increases with more puncture attempts [9]. The current study consistently showed a similar relationship between increasing puncture attempts and increased risk of early complications. Still, no such relationship occurred between puncture number and late complication incidence. In the literature, no conclusive evidence exists in terms of complications happening between breast surgery and TIVAD implantation. Only opinions suggesting no significant effect have been reported. Similarly, in our study, there was no significant association between complications and whether or not the patient had undergone breast-conserving surgery or mastectomy. In some studies on TIVAD implantation in BC patients, the tumor side considered in the selection of the TIVAD implantation side opposite to the tumor is preferred. In our study group, the right-sided SCV was the preferred route, and there was no association between the side of the BC and complications. There is also little or no consensus on whether there is an association between TIVAD implantation and patients undergoing axillary dissection or receiving adjuvant radiotherapy [10,11]. The present study did not show any effect of axillary dissection or radiotherapy on complications from TIVAD implantation.

To evaluate catheter-related infections, which were the most common complication in our BC patients with TIVAD, in a study including all disease groups and all implantation routes, the infection rate due to TIVAD implantation was reported as 6.6% [12]. Another study that included all disease groups reported the infection rate as 14% when the SCV route was used [13]. In a study of 2996 BC patients who underwent TIVAD implantation using different routes, the incidence of infection was 1.6%, and the rate of requiring removal due to this infection was 0.8%. However, this study did not investigate further potential risks for infection [14]. In our group, ten (1.9%) patients developed catheter-related infections, and the comorbidity and smoking history of the patients may have been the cause of infection. Port removal was required in five (0.9%) patients due to catheter-related infection.

In another study, after loco-regional treatment of BC, especially in the early period, there was an increase in pain in the breast, arm, and shoulder and severe limitation in functional capacity [15]. In another study, pain after breast surgery occurred at a rate of 30%–50%, limitation of arm and shoulder movement at 35%, and lymphedema at 15%–25% [16]. In our study, there were similar rates of late effects specific to BC surgery, but there was no evidence that TIVAD implantation increased these rates.

This study has some limitations, mainly due to its retrospective design and low number of patients. No criteria were set for the placement side of TIVAD by assessing the BC side. Furthermore, the study did not examine the effects after infusion of high concentrations of nutritional products that patients could have potentially used and that may have caused complications. However, this study remains pertinent and useful as a source of real-life data. In many medical centers, TIVAD is implanted by following the right-sided SCV route without considering the BC side, which is preferred because it is thought not to increase complication rates. Although the catheter-related infection rate is high in patients undergoing radiotherapy, the low number of patients whose ports were removed confirms this preference. Similarly, implanting TIVAD on the side of axillary dissection does not increase the complication rate.

The importance of systemic treatment of BC has increased in recent years. The application of targeted therapies necessitates the need for long-term intravenous access. Therefore, the need for ports has become increasingly important in patients with BC. In addition, the fact that all patients in this study had completed surgical treatment and that TIVADs were implanted via the right-sided SCV route in almost all of them is valuable in terms of standardization. In this respect, our study is worthwhile because the need for port catheters will be more frequent in patients needing neoadjuvant treatment. This aspect of the present study will pave the way for prospective studies.

In conclusion, this study demonstrated that the right-sided SCV route in TIVAD implantation is a safe approach in all BC patients, regarding the lower risk of complications related to implantation and not changing the rates of BC-specific late effects. The study also showed that further experience and US use will reduce the risk of complications, especially early ones. The study can only suggest that for patients scheduled for postoperative radiotherapy and who have additional comorbidities, clinicians may prefer implantation using another route to prevent catheter-related infection risks.

## Figures and Tables

**Table 1:** Demographic and clinical data of the patients.

Right subcvlavian vein n = 524		n	%
**Mean age (SD) in years, range**		56.1 (±11.4)	(28–81)
**Mean body mass index (SD), range**		25.3 (±6.5)	(19–38)
**Cancer side**	**Right**	280	53.4
	**Left**	244	46.6
**Insertion method**	**Anatomic landmark**	96	18.3
	**Ultrasound**	428	81.7
**Number of punctures**	**1**	182	34.7
	**2**	214	40.8
	**≥3**	128	24.5
**Surgery**	**BCS**	351	66.9
	**Mastectomy**	173	33.1
**Axilla**	**AD**	106	20.2
	**SLNB/left-sided cancer**	418	79.8
**Radiotherapy**	**No**	345	65.8
	**Yes**	179	34.2
**Early complications**		12	2.3
	**Arterial puncture**	7	1.4
	**Pneumothorax**	3	0.6
	**Malposition**	2	0.3
**Late complications**		14	2.6
	**Catheter-related infection**	10	1.9
	**Thrombosis**	3	0.6
	**Catheter fracture**	1	0.2
**Overall complications**		26	4.9
**Port removal**		8	1.5

BCS: breast-conserving surgery; AD: axillary dissection; SLNB: sentinel lymph node biopsy; SD: standard deviation.

**Table 2:** Risk factor analysis for complications.

Right subclavian vein		Early complications		Late complications		Overall complications	
		n (%)	p	n (%)	p	n (%)	p
**Mean age, (complication no/yes)**		55.9/57.2	0.06	56.0/56.5	0.34	55.9/56.7	0.24
**Mean body mass index, (Complication no/yes)**		25.4/23.8	0.08	25.2/26.3	0.23	25.4/24.5	0.32
**Cancer side**	**Right**	7 (2.5%)	0.48	10 (3.6%)	0.13	17 (6.1%)	0.14
	**Left**	5 (2.0%)		4 (1.6%)		9 (3.7%)	
**Insertion method**	**Anatomic landmark**	6 (6.3%)	0.012	3 (3.1%)	0.48	9 (9.4%)	0.032
	**Ultrasound**	6 (1.4%)		11 (2.6%)		17 (4.0%)	
**Number of punctures**	**1**	2 (1.1%)	0.021	4 (2.2%)	0.26	6 (3.3%)	0.011
	**2**	3 (1.4%)		4 (1.9%)		7 (3.4%)	
	**≥ 3**	7 (5.5%)		6 (4.7%)		13 (10.7%)	
**Surgery**	**BCS**	7 (2.0%)	0.36	10 (2.8%)	0.48	17 (4.8%)	0.51
	**Mastectomy**	5 (2.9%)		4 (2.3%)		9 (5.2%)	
**Axilla**	**AD**	4 (3.8%)	0:21	5 (4.7%)	0.13	9 (8.5%)	0.06
	**SLNB/Left-sided cancer**	8 (1.9%)		9 (2.2%)		17 (4.1%)	
**Radiotherapy**	**No**	8 (2.3%)	0.61	6 (1.7%)	0.06	14 (4.1%)	0.13
	**Yes**	4 (2.2%)		8 (4.5%)		12 (6.7%)	

BCS: breast-conserving surgery; AD: axillary dissection; SLNB: sentinel lymph node biopsy.

**Table 3:** Analyses on catheter-related infections.

Catheter-related infection		Univariate			Multivariate			
						95% CI EXP(B)		
		No n = 514	Yes n = 10	p	OR	Lower	Upper	p
**Mean age (SD)**		55.9 (± 11.1)	56.4 (± 12.8)	0.12	1.34	0.84	1.76	0.23
**Mean body mass index (SD)**		25.3 (± 7.2)	26.2 (± 6.1)	0.10	1.54	0.82	2.18	0.14
**Cancer side**	**Right**	272 (97.1%)	8 (2.9%)	0.081				
	**Left**	242 (99.2%)	2 (0.8%)		1.85	0.75	6.92	0.11
**Insertion method**	**Anatomic landmark**	93 (96.9%)	3 (3.1%)	0.27				
	**Ultrasound**	421 (98.4%)	7 (1.6%)		1.79	0.49	5.14	0.34
**Number of punctures**	**1**	180 (98.9%)	2 (1.1%)	0,16				
	**2**	211 (98.6%)	3 (1.4%)		1.28	0.79	3.46	0.48
	**≥ 3**	123 (96.1%)	5 (3.9%)		2.47	0.82	6.51	0.12
**Surgery**	**BCS**	343 (97.7%)	8 (2.3%)	0.30				
	**Mastectomy**	171 (98.8%)	2 (1.2%)		1.99	0.42	5.66	0.38
**Axilla**	**AD**	103 (97.2%)	3 (2.8%)	0.32				
	**SLNB/Left-sided cancer**	411 (98.3%)	7 (1.7%)		1.71	0.43	6.72	0.44
**Adjuvant radiotherapy**	**No**	342 (99.1%)	3 (0.9%)	0.022				
	**Yes**	172 (96.1%)	7 (3.9%)		4.64	1.18	12.16	0.028

BCS: breast-conserving surgery; AD: axillary dissection; SLNB: sentinel lymph node biopsy, SD: standard deviation.
